# Modeling of cell cultivation for monoclonal antibody production processes considering lactate metabolic shifts

**DOI:** 10.1002/btpr.3486

**Published:** 2024-06-24

**Authors:** Kozue Okamura, Sara Badr, Yusuke Ichida, Akira Yamada, Hirokazu Sugiyama

**Affiliations:** ^1^ Department of Chemical System Engineering The University of Tokyo Tokyo Japan

**Keywords:** Chinese hamster ovary cells, dissolved oxygen, glutamine, lactate metabolic shifts, mechanistic model, orbitally‐shaken tanks, process design

## Abstract

Demand for monoclonal antibodies (mAbs) is rapidly increasing. To achieve higher productivity, there have been improvements to cell lines, operating modes, media, and cultivation conditions. Representative mathematical models are needed to narrow down the growing number of process alternatives. Previous studies have proposed mechanistic models to depict cell metabolic shifts (e.g., lactate production to consumption). However, the impacts of variations of some operating conditions have not yet been fully incorporated in such models. This paper offers a new mechanistic model considering variations in dissolved oxygen and glutamine depletion on cell metabolism applied to a novel Chinese hamster ovary (CHO) cell line. Expressions for the specific rates of lactate production, glutamine consumption, and mAb production were formulated for stirred and shaken‐tank reactors. A deeper understanding of lactate metabolic shifts was obtained under different combinations of experimental conditions. Lactate consumption was more pronounced in conditions with higher DO and low glutamine concentrations. The model offers mechanistic insights that are useful for designing advanced operation strategies. It can be used in design space generation and process optimization for better productivity and product quality.

## 1 INTRODUCTION

Monoclonal antibody (mAb) drugs are becoming increasingly important in the fight against debilitating diseases, such as cancer and autoimmune diseases, with many new applications on the horizon.^1^, ^2^ Chinese hamster ovary (CHO) cells are a popular choice for the cell cultivation step in mAb production due to the maturity of technologies such as transgene amplification.^3^ To meet the increasing mAb demand, there is a growing number of alternatives for optimizing production, such as modifying host cell lines,^4^ operating modes (e.g., perfusion), media,^5^ cultivation conditions (e.g., pH,^6^ temperature,^7^ and dissolved oxygen (DO) levels^8^). Building comprehensive and representative mathematical models can greatly help to narrow down process candidates to effective combinations of process alternatives, which is crucial for reducing the number of experiments and the associated costs.^9^

**FIGURE 1:**
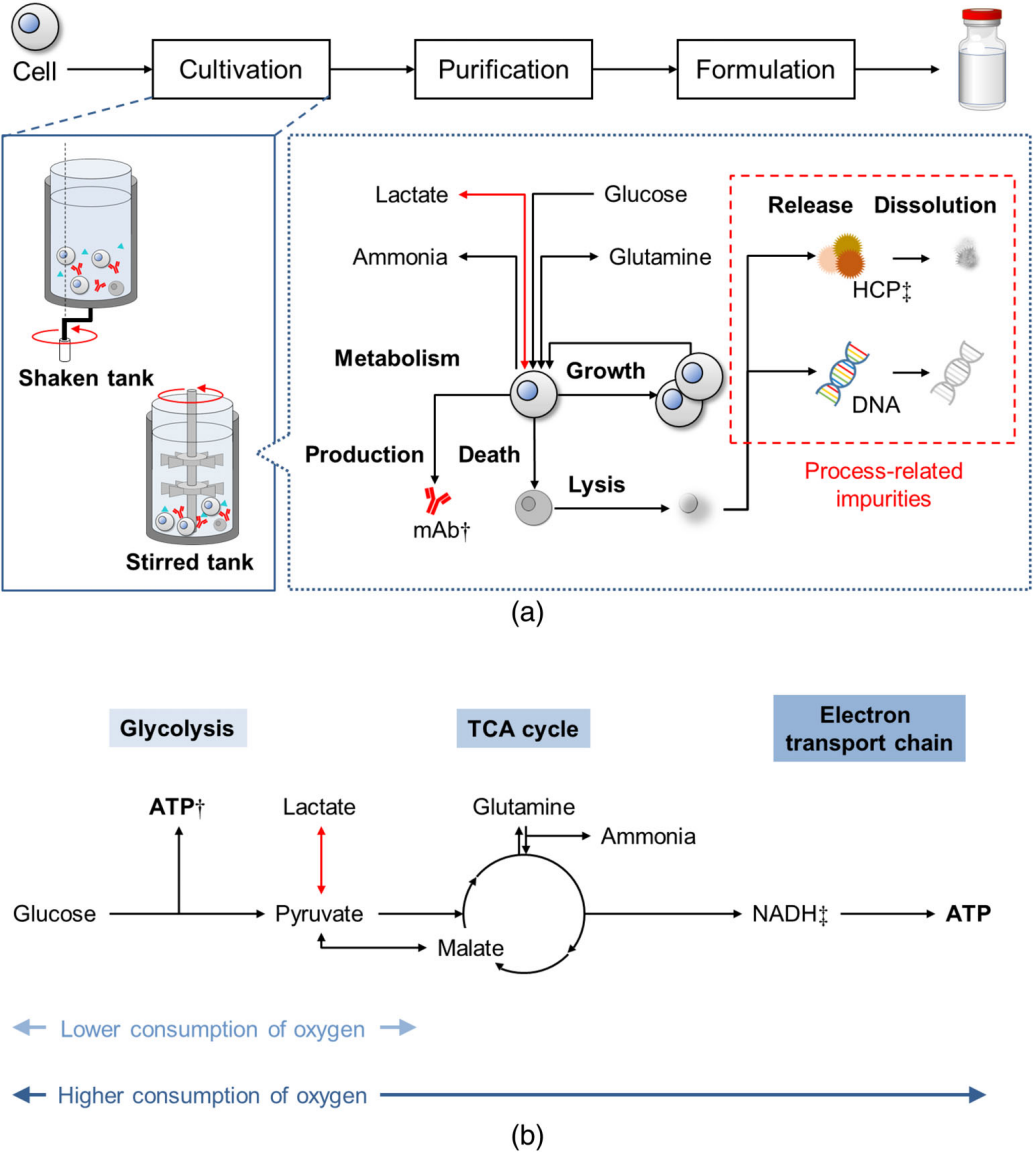
(a) A typical monoclonal antibody (mAb) production process and cell cultivation process modeled in this work. †: Monoclonal antibody, ‡: Host cell protein., (b) Relevant metabolic pathways. †: Adenosine triphosphate, ‡: Nicotinamide adenine dinucleotide.

Figure 1a shows a typical mAb production process and the cell cultivation phenomena modeled in this work. During cultivation, cells proliferate and produce mAb while consuming nutrients (e.g., glucose, glutamine) and generating metabolites (e.g., lactate, ammonia). Some of the metabolites affect cell death and performance, for example, the reported inhibiting effect of lactate on cell growth and cell viability.^10^ After cell death and lysis, some process‐related impurities are released, which could have an impact on the product quality, such as host cell protein (HCP) affecting product fragmentation and aggregation.^11^ Therefore, modeling the cell death and generated impurity profiles with high accuracy is essential for process design. Accurate modeling of lactate concentrations has been shown to be critical for correctly predicting cell death and the subsequent impurity generation.^12^ The accurate estimation of lactate concentrations is also important for pH prediction and osmolality control, which allows for the implementation of more efficient nutrient‐feeding strategies.^13^

Shifts in lactate metabolic behavior can occur, for example, where cells switch from net lactate production to net consumption.^14^ Previous research studies have suggested potential causes and mechanisms of the shifts.^14^, ^15^, ^16^, ^17^, ^18^, ^19^, ^20^ Figure 1b shows a schematic diagram of the relevant metabolic pathways. Possible triggers or correlated factors include glucose or glutamine deficiency, which might cause lactate consumption to keep supplying pyruvate under conditions where cells have higher oxygen consumption rates. Other factors include low temperatures in the reactor, low pCO_2_ levels, or base addition for pH adjustment. Shifts between lactate production to consumption can impede the accurate prediction of lactate concentrations, especially with the complexity of the causes of such shifts.

Traditionally, existing unstructured mechanistic models^21^, ^22^ rely on constant model parameters for cell specific consumption or production rates under certain cultivation conditions.^23^ Thus, such models cannot follow the shifts in lactate metabolic behavior. In order to compensate for this gap, there are data‐driven models,^24^, ^25^ and hybrid modeling approaches that dynamically update the production/consumption rates with changes in process conditions.^12^, ^26^ On the other hand, there are still advantages to model the process mechanistically in a simple way for practical use. Mechanistic models are more suitable modeling methods for extrapolations, offer higher interpretability to relationships between inputs and outputs, and require less data for training than data‐driven counterparts.

Some research studies have proposed mechanistic models to address the shift from lactate production to consumption. Alhuthali & Kontoravdi^27^ developed a population balance model to predict impurity release from cells. The model used a variable lactate production term as a function of glucose consumption and a constant lactate consumption term. Wang et al.^28^ developed a kinetic model with two sets of parameter values to consider the impact of temperature shifts on the lactate metabolism. In this model, the lactate consumption term became larger after a downward temperature shift. While kinetic models consider concentrations of components outside the cell, stoichiometric models focus on detailed metabolic fluxes inside the cells, which allows the consideration of more components and more complex reactions albeit at steady state conditions.^29^ Integrated models combining stoichiometric and kinetic models attempt to retain the high resolution of the former in addition to the simplicity and dynamic representation of the latter. However, they can be more difficult to validate and have large computational requirements. Integrated models have been presented to describe metabolic lactate shifts as a function of temperature and/or pH shifts.^29^, ^30^, ^31^ The impact of other operating conditions such as changes in DO levels are yet to be elucidated.

In this work, we developed a kinetic cultivation model incorporating lactate shifts with its impacts on cell growth, death, and impurity generation. The model considers the impact of glutamine depletion under different oxygen environments in relation to the lactate metabolic shifts. Alternative formulations were proposed for critical variable parameters under different combinations of culture conditions, and the cultivation model was updated accordingly. The work investigates the impact of changes in reactor types and design on the process performance. The model was developed using cultivation data of the novel high‐productivity CHO‐MK cell line,^32^ which often displays lactate metabolic shifts. Since the model includes mAb production and impurity generation, it can be used for process performance evaluation in terms of both productivity and quality.

## 2 METHODS

### 2.1 Cell culture data acquisition

**FIGURE 2:**
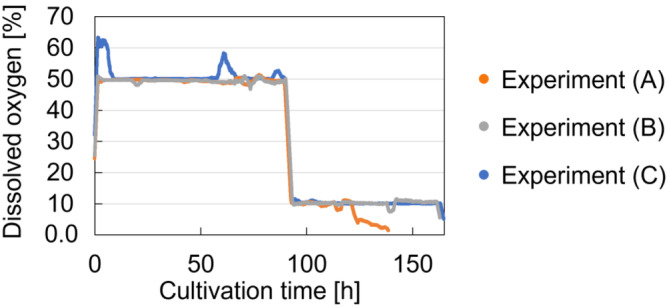
Observed dissolved oxygen profiles.

Cell culture data used for model development and validation has been obtained from an existing pool of experimental data from the Kobe GMP consolidated lab of the Manufacturing Technology Association of Biologics in Japan. Data from three experiments with the newly developed high‐productivity CHO‐MK 9E‐1 cell line^32^ have been used. Compared to the conventional CHO‐K1 cell line, the CHO‐MK cell line has: (1) Shorter doubling time (<10 h) and 3‐ to 5‐fold higher achieved viable cell density; (2) Higher rates of antibody production, especially in the early phase of culture, (3) Antibody production as high as 5 g L^−1^ within 5 days by controlling the dissolved oxygen concentration.^32^ All the experiments featured fed‐batch cultivation. Proprietary media was obtained from FUJIFILM Wako Pure Chemical Corporation. Glucose was available in the basal media at a concentration of 9.6 g L^−1^ and was added to the feed media to reach a concentration of 120 g L^−1^ in the feed. While, 4 mmol L^−1^ of glutamine were added to the basal media without further additions in the feed media. Experiments (A) and (B) used a 50 L orbitally‐shaken tank (hereafter referred to as shaken tank) with a single‐use bag (Fujimori Kogyo). Experiment (B) was conducted in conditions similar to those of Experiment (A). However, some differences existed between the two. For example, Experiment (B) was run for a longer duration and the timing of the feed differed between the two experiments. DO was set to be the same for both experiments, however during Experiment (A), DO levels could not be maintained in the final stage of the run before the experiment was terminated. Experiment (C) used a 50 L stirred tank with mixing blades and a single‐use bag (Cytiva). Table 1 shows a summary of the experimental conditions. The seeding density for Experiments (A), (B), and (C) were 2.33 × 10^6^ cells mL^−1^, 2.32 × 10^6^ cells mL^−1^, and 2.45 × 10^6^ cells mL^−1^, respectively. The feed was added in a continuous manner after day 2 to ensure sufficient amounts of nutrients in the culture environment. The values displayed in Table 1 represent the feed flow rate per day. The relative dissolved oxygen concentration to the saturation concentration at 37 °C (hereafter referred to as DO) is an important factor that can affect both cell viability and the product quality attributes.^33^ A balance is required between DO levels to maintain normal metabolism, while avoiding the extra burden of producing antioxidants and the harmful effect of excessive reactive oxygen species.^33^, ^34^ Based on the recommendation of the cell line developers, the DO setpoint was shifted from 50% to 10% to suppress the growth phase. The change was set to take place on the fourth day of culture if the viable cell density had reached 50 × 10^6^ cells mL^−1^. Figure 2 shows the observed DO profiles. Daily offline measurements of viable cell density, glucose, glutamine, lactate, ammonia, HCP, and DNA concentrations were available. As an exception, glutamine was not measured in Experiment (B). Dead cell density was calculated from the viable cell density and viability, which was also measured offline. Online measurements were available for DO, air flow rate into the gas phase, oxygen sparging rate, agitation rate, pH, solution volume, and temperature in the reactors at 1‐min intervals. A list of the devices used for measurements is available in Table S1 in the **Supporting Information**.

**TABLE 1:** Experimental conditions. [Correction added after first online publication on 30 July 2024. Table 1 has been Updated.]

Experiment	(A)	(B)	(C)
Operation mode	Fed‐batch		
Cell line	CHO‐MK 9E‐1		
Product	MAB1		
Reactor type	Orbitally‐shaken tank	Orbitally‐shaken tank	Stirred tank
Reactor volume [L]	50		
Initial solution volume [L]	30		
Experiment duration [h]	139 (6 days)	164 (7 days)	164 (7 days)
Measured dissolved oxygen range [%]	0 h–92 h: 50	0 h–92 h: 50	0 h–92 h: 50
	92 h–: 10	92 h–: 10	92 h–: 10
	(After 120 h, there was a gradual decrease to 1.5%)		
Measured pH range [−]	6.9–7.4	6.8–7.4	6.8–7.1
Measured temperature range [°C]	36.3–37.3	36.9–37.3 (Bigger fluctuations in temperature were observed before 27 h to ~34.7 °C)	36.5–37.2
Agitation rate [rpm]	67–80	65–80	100
Gas phase air flow [L h^−1^]	30–120	30–120	56–58
Oxygen sparging rate [L h^−1^]	0–60	0–50	0–50
Glucose concentration in feed media [g L^−1^]	120		
Feeding strategy [% day^−1^] (Compared to initial solution volume)	Day 2: 5	Day 2: 5	Day 2: 5
	Day 3–: 10	Day 3–4: 10	Day 3–4: 10
		Day 5–: 12	Day 5–: 12
Final solution volume [L]	40.6	45.0	45.2

### 2.2 Model development

#### 2.2.1 Mass balance equations

The model presented here was developed based on models proposed by previous research work.^21^, ^22^ Equations ((1), (2), (3), (4), (5), (6), (7)) show the mass balance equations of the main components related to fundamental cell metabolism: viable cell density, concentrations of mAb, glucose, glutamine, lactate, and ammonia, and solution volume.

(1) $$\frac{d\left(VX_{v}\right)}{\mathrm{dt}}=\left(\mu -\mu_{d}\right)VX_{v}-F_{\text{bleed}}X_{v}$$

(2) $$\frac{d\left(\mathrm{VP}\right)}{\mathrm{dt}}=Q_{p}VX_{v}-\left(F_{\text{harvest}}+F_{\text{bleed}}\right)P$$

(3) $$\frac{d\left(V\left[\mathrm{GLC}\right]\right)}{\mathrm{dt}}=\begin{array}{l} -\left(\frac{\mu }{Y_{X_{v}/\mathrm{glc}}}+m_{\mathrm{glc}}\right)VX_{V}+F_{\text{in}}c_{\text{in}\_\mathrm{glc}}+F_{\text{suppl}}c_{\text{suppl}}\\ -\;\left(F_{\text{harvest}}+F_{\text{bleed}}\right)\left[\mathrm{GLC}\right] \end{array}$$

(4) $$\frac{d\left(V\left[\mathrm{GLN}\right]\right)}{\mathrm{dt}}=-Q_{\mathrm{gln}}VX_{v}+F_{\text{in}}c_{\text{in}\_\mathrm{gln}}-\left(F_{\text{harvest}}+F_{\text{bleed}}\right)\left[\mathrm{GLN}\right]$$

(5) $$\frac{d\left(V\left[\mathrm{LAC}\right]\right)}{\mathrm{dt}}=Q_{\mathrm{lac}}VX_{v}-\left(F_{\text{harvest}}+F_{\text{bleed}}\right)\left[\mathrm{LAC}\right]$$

(6) $$\frac{d\left(V\left[\mathrm{AMM}\right]\right)}{\mathrm{dt}}=\left(k_{\mathrm{amm}1}Q_{\mathrm{gln}}+k_{\mathrm{amm}2}\right)VX_{v}-\left(F_{\text{harvest}}+F_{\text{bleed}}\right)\left[\mathrm{AMM}\right]$$

(7) $$\frac{d\left(V\right)}{\mathrm{dt}}=F_{\text{in}}+F_{\text{suppl}}-\left(F_{\text{harvest}}+F_{\text{bleed}}\right)$$

In the above equations, $X_{v}$ is the viable cell density, and V is the solution volume. μ is the specific cell growth rate, $\mu_{d}$ is the specific cell death rate, while $\mu_{\max}$ and $k_{d}$ are their maximum values. $F_{\text{in}}$ is the inlet flowrate. $F_{\text{suppl}}$ is the supplementary glucose flowrate. $k_{\mathrm{amm}1}$ and $k_{\mathrm{amm}2}$ are constants. It should be noted that the term $k_{\mathrm{amm}1}Q_{\mathrm{gln}}$ in Equation (6) is only applied for positive values of $Q_{\mathrm{gln}}$ (i.e., net glutamine consumption phases), otherwise the term is neglected. $F_{\text{bleed}}$ and $F_{\text{harvest}}$ are the bleed and harvest flowrates, respectively. These terms describe perfusion mode cultivation. In this work, all the experiments were in fed‐batch mode; therefore, these terms were set to 0 in the calculation.

Equations ((8), (9), (10)) show the mass balance equations of the main components related to impurity generations: dead cell density, and concentrations of HCP and DNA. Okamura, et al.^12^ has shown that the HCPs were mainly generated from the lysis of dead cells and that the generation from viable CHO‐MK cells was relatively negligible under similar experimental conditions as in this work. The same assumption was used in this model.

(8) $$\frac{d\left({\mathrm{VX}}_{d}\right)}{\mathrm{dt}}=\mu_{d}VX_{v}-\frac{r_{\text{dcell}}{\mathrm{VX}}_{d}}{K_{\text{ldcell}}+X_{d}}-F_{\text{bleed}}X_{d}$$

(9) $$\frac{d\left(V\left[\mathrm{HCP}\right]\right)}{\mathrm{dt}}=\begin{array}{l} Y_{\mathrm{hcp}/X_{d}}\left(\frac{r_{\text{dcell}}{\mathrm{VX}}_{d}}{K_{\text{ldcell}}+X_{d}}\right)-\left(\frac{r_{\mathrm{hcp}}V\left[\mathrm{HCP}\right]}{K_{\text{dhcp}}+\left[\mathrm{HCP}\right]}\right)\\ -\;\left(F_{\text{bleed}}+F_{\text{harvest}}\right)\left[\mathrm{HCP}\right] \end{array}$$

(10) $$\frac{d\left(V\left[\mathrm{DNA}\right]\right)}{\mathrm{dt}}=\begin{array}{l} Y_{\mathrm{dna}/X_{d}}\left(\frac{r_{\text{dcell}}{\mathrm{VX}}_{d}}{K_{\text{ldcell}}+X_{d}}\right)-\left(\frac{r_{\mathrm{dna}}V\left[\mathrm{DNA}\right]}{K_{\text{ddna}}+\left[\mathrm{DNA}\right]}\right)\\ -\;\left(F_{\text{bleed}}+F_{\text{harvest}}\right)\left[\mathrm{DNA}\right] \end{array}$$

In the above equations, $X_{d}$ is the dead cell density. $\left[\mathrm{HCP}\right]$ and $\left[\mathrm{DNA}\right]$ are HCP, and DNA concentrations, respectively. $r_{\text{dcell}}$, $r_{\mathrm{hcp}}$, and $r_{\mathrm{dna}}$ are the rates of dead cell lysis, HCP, and DNA dissolution, respectively. $K_{\text{ldcell}}$, $K_{\text{dhcp}}$, and $K_{\text{ddna}}$ are the lysis and dissolution constants, respectively. $Y_{\mathrm{hcp}/X_{d}}$ and $Y_{\mathrm{dna}/X_{d}}$ are the constants describing the specific mass released from a dead cell of HCP and DNA, respectively.

This work investigated the variation in $Q_{\mathrm{gln}}$, $Q_{\mathrm{lac}}$, and $Q_{p}$, which can change according to operating conditions. These three parameters can be experimentally determined from the available data. Their variability along the experimental runs was analyzed to better understand the underlying factors contributing to their variations. The following sections discuss the variation of these parameters over time and their correlation with other factors.

#### 2.2.2 Parameter determination from experimental data

##### Specific rates of lactate production and glutamine consumption

**FIGURE 3:**
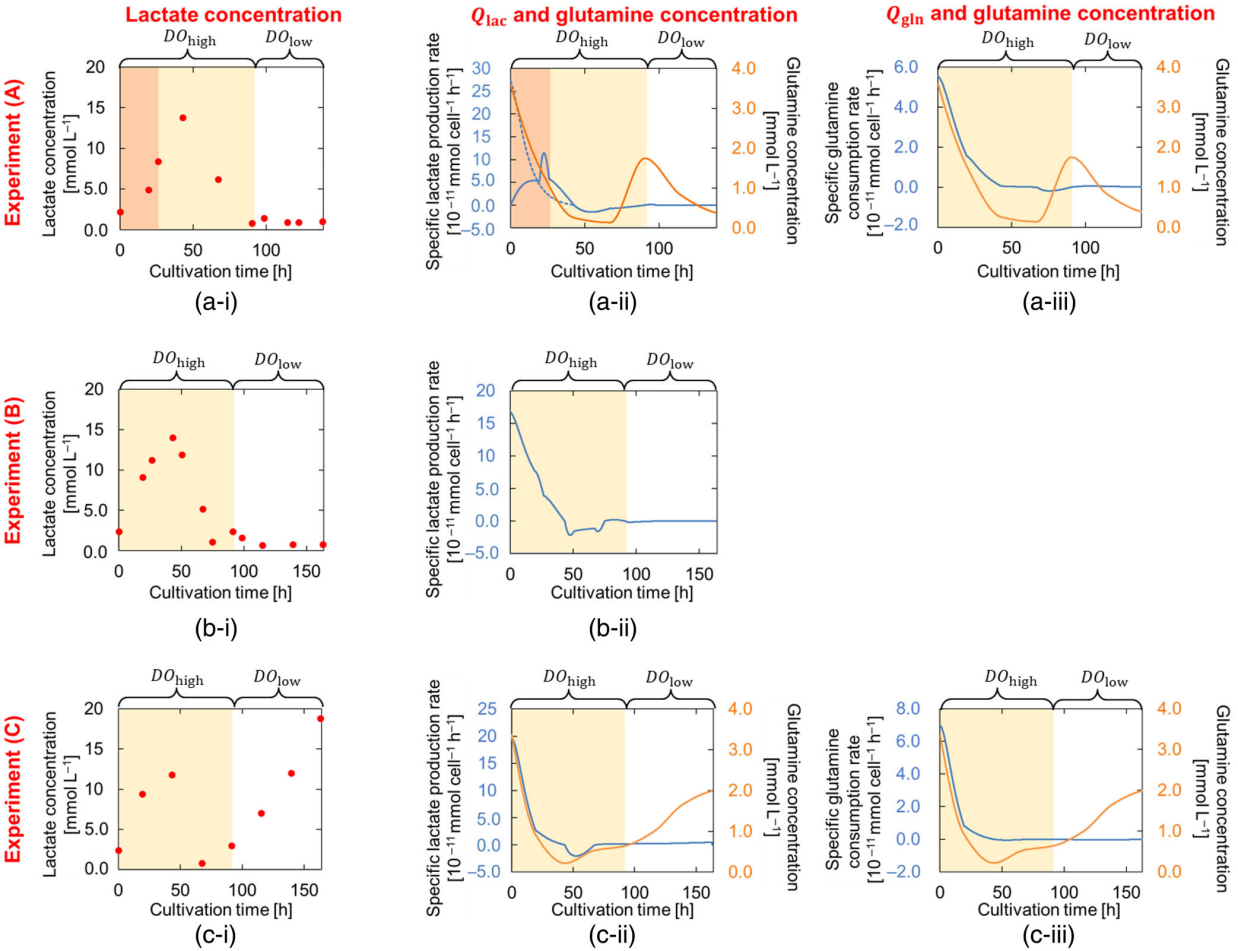
Lactate and glutamine concentration profiles and experimentally determined model parameters for (a) Experiment (A), (b) Experiment (B), and (c) Experiment (C). On the left (i) offline measurements of lactate concentrations. In the middle (ii) calculated specific lactate production rates $Q_{\mathrm{lac}}$ and interpolated glutamine concentrations. On the right (iii) calculated specific glutamine consumption rates $Q_{\mathrm{gln}}$ and interpolated glutamine concentrations. Glutamine concentrations were not available for Experiment (B), and thus $Q_{\mathrm{gln}}$ were only estimated and not determined experimentally. Shaded areas represent high DO (${\mathrm{DO}}_{\text{high}}$) levels (~50%). In Experiment (A), orange‐shaded areas represent a region where $Q_{\mathrm{lac}}$ showed behavior different from Experiments (B) and (C). Differences could be attributed to inaccuracies in lactate measurements or their timing. The dotted line in (a‐ii) shows $Q_{\mathrm{lac}}$ calculated when measurements taken on day one were neglected. The dotted line shows similar behavior to Experiments (A) and (C).

The measured viable cell density, lactate, and glutamine concentrations were interpolated using the Piecewise Cubic Hermite Interpolating Polynomial (PCHIP) method. The specific rates of lactate production $Q_{\mathrm{lac}}$ and glutamine consumption $Q_{\mathrm{gln}}$ were calculated by using the interpolated values and the simulated solution volume with Equations (4) and (5), respectively. Figure 3 shows the observed lactate concentration profiles and the calculated $Q_{\mathrm{lac}}$ and $Q_{\mathrm{gln}}$ for Experiments (A), (B), and (C). Figure 3 **(ii)** and **(iii)** show high variabilities along the duration of the run in the values of $Q_{\mathrm{lac}}$ and $Q_{\mathrm{gln}}$, respectively. This supports the need for achieving a dynamic representation of these parameters in the model. Different trends were observed between $Q_{\mathrm{lac}}$ and $Q_{\mathrm{gln}}$ and the glutamine concentrations at each DO level. This is consistent with the work of Farzan, et al.^35^ which showed variations in specific consumption and production rates of different nutrients and metabolites with varying DO levels. As shown in Figure 3, at higher DO levels (shaded in yellow), $Q_{\mathrm{lac}}$ and $Q_{\mathrm{gln}}$ decreased as glutamine levels dropped. $Q_{\mathrm{gln}}$ stabilized at around 0 mmol cell^−1^ h^−1^ as a certain threshold of glutamine concentrations was reached (~1 mmol L^−1^), indicating no further glutamine consumption. Below this level, the specific lactate production rate slowed, followed by a phase of lactate consumption. At low DO levels, $Q_{\mathrm{lac}}$ and $Q_{\mathrm{gln}}$ became insensitive to changes in the glutamine levels, even at high glutamine concentrations. For Experiment (A), a different shape of the $Q_{\mathrm{lac}}$ variation was observed on the first day of cultivation. $Q_{\mathrm{lac}}$ exhibited a sharp peak in value (shaded in orange in Figure 3(a‐ii)). The measured lactate concentrations (see Figure 3(a‐i)) exhibited sharper changes in the slope between the different measurements than in the other two experiments, where the rate of increase in lactate concentrations slowed down before going into the lactate consumption phase. The observed deviations in the slope in Experiment (A) could have resulted from small inaccuracies in the reported times when the measurements were taken or from slight experimental errors in the measurement of lactate concentrations. The dotted line in Figure 3(a‐ii) shows the change in the specific lactate production rates by carrying out the interpolation neglecting the measurements taken on the first day after the start of the experiment. The recalculated $Q_{\mathrm{lac}}$ (dotted line) shows very similar behavior to that exhibited in Experiments (B) and (C) with a similar correlation to the glutamine concentrations as shown in Experiment (C). Therefore, it can be suggested that the deviation in Experiment (A) came from an experimental error rather than differences in the setups of the experiments or reaction mechanism.

##### Specific mAb production rate

**FIGURE 4:**
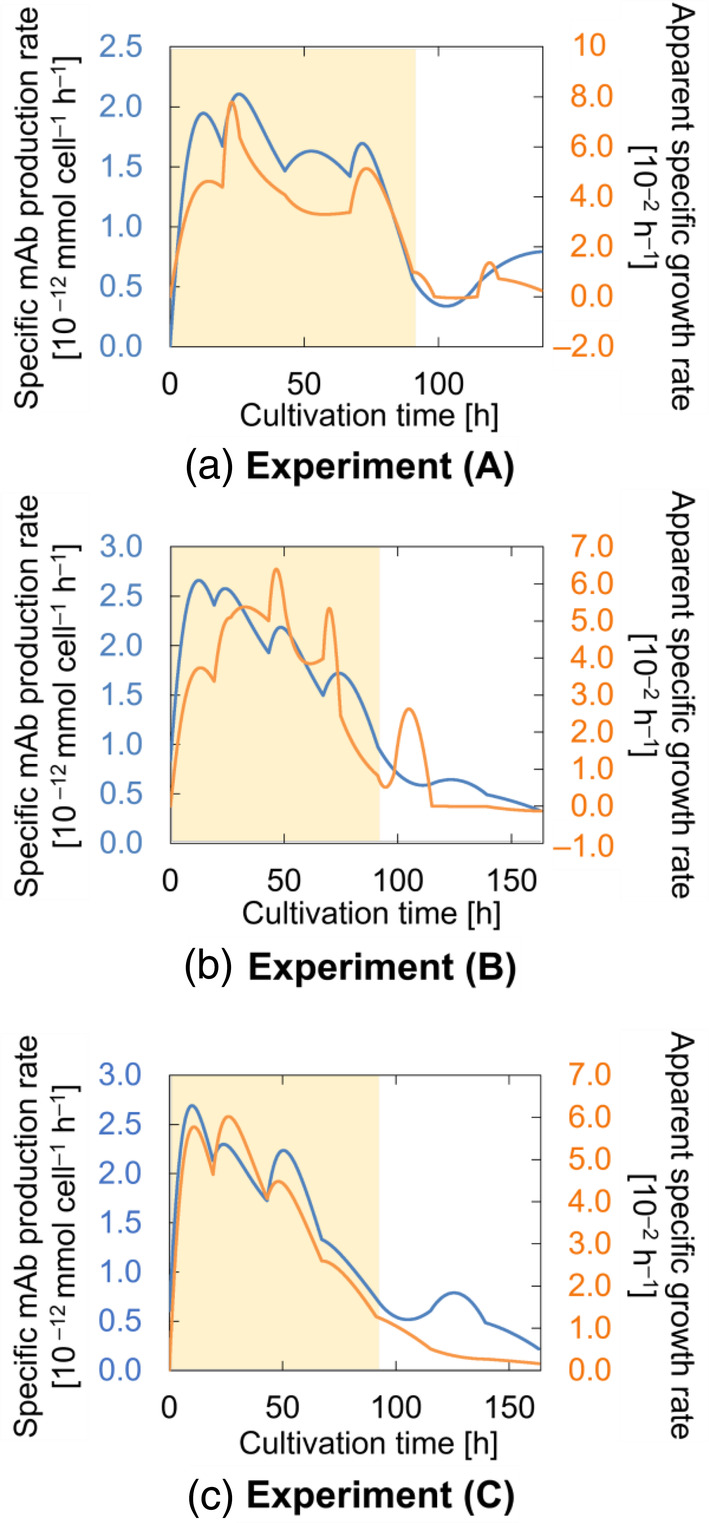
The specific mAb production rate $Q_{p}$ (blue lines) and apparent specific growth rate $\left(\mu -\mu_{d}\right)$ (orange lines) profiles for (a) Experiment (A), (b) Experiment (B), and (c) Experiment (C). Shaded areas represent high dissolved oxygen (${\mathrm{DO}}_{\text{high}}$) levels.

The specific mAb production rate $Q_{p}$ was calculated by substituting the interpolated observed experiment data (i.e., mAb concentrations) and the calculated solution volume into Equation (2). The apparent specific growth rate $\left(\mu -\mu_{d}\right)$ was calculated in the same way using Equation (1) and the interpolated observed viable cell density. Figure 4 shows the profiles of calculated $Q_{p}$ and $\left(\mu -\mu_{d}\right)$ for each experiment. The figure confirms the varying nature of $Q_{p}$ and the correspondence of the variations with those observed in $\left(\mu -\mu_{d}\right)$ throughout the experimental run (except for the later phases, where a tradeoff between apparent growth and productivity was observed). Also, it was confirmed that changes in DO and $Q_{p}$ levels were concurrent.

#### 2.2.3 Formulation of the variable parameters and their implementation

Based on the previous observations, alternative formulations were proposed for $Q_{\mathrm{lac}}$, $Q_{\mathrm{gln}}$, and $Q_{p}$ to describe the expected variations as shown in Equations ((11), (12), (13)). At high DO levels, a sigmoid function was used to describe the dependence of $Q_{\mathrm{lac}}$ and $Q_{\mathrm{gln}}$ on glutamine concentrations. Such a function describes the effects at low glutamine concentration levels, where the correlation between glutamine concentration levels and the $Q_{\mathrm{lac}}$ and $Q_{\mathrm{gln}}$ is weaker (Figure 3 **(ii)** and **(iii)** yellow shaded areas). This model fits the hypothesis shown in Figure 1b that cells switch from glutamine consumption to lactate consumption at lower concentrations of glutamine. At low DO levels, lactate production and glutamine consumption were observed to be less sensitive to variations in glutamine concentrations. Therefore, constant values for $Q_{\mathrm{lac}}$ and $Q_{\mathrm{gln}}$ were assumed in Equations (11) and (12) at low DO levels (Figure 3 **(ii)** and **(iii)**).

(11) $$Q_{\mathrm{lac}}=\left\{\begin{array}{c} {\left.\frac{k_{\mathrm{lac}11}}{k_{\mathrm{lac}12}+\exp\left\{-k_{\mathrm{lac}13}\left[\mathrm{GLN}\right]\right\}}+k_{\mathrm{lac}14}\;\right|}_{\mathrm{DO}={\mathrm{DO}}_{\text{high}}}\\ {\left.k_{\mathrm{lac}21}\;\right|}_{\mathrm{DO}={\mathrm{DO}}_{\mathrm{low}}} \end{array}\right.$$

(12) $$Q_{\mathrm{gln}}=\left\{\begin{array}{c} {\left.\frac{k_{\mathrm{gln}11}}{k_{\mathrm{gln}12}+\exp\left\{-k_{\mathrm{gln}13}\left[\mathrm{GLN}\right]\right\}}+k_{\mathrm{gln}14}\;\right|}_{\mathrm{DO}={\mathrm{DO}}_{\text{high}}}\\ {\left.k_{\mathrm{gln}21}\;\right|}_{\mathrm{DO}={\mathrm{DO}}_{\mathrm{low}}} \end{array}\right.$$

(13) $$Q_{p}=q_{p}\left(\mu -\mu_{d}\right)$$

In Equations (11) and (12), $k_{\mathrm{gln}}$ and $k_{\mathrm{lac}}$ are constants. At higher DO levels, two separate terms were used to represent consumption and production. Lactate production and glutamine consumption were assumed to be a function of glutamine concentrations at high DO levels. At low DO levels, Equations (11) and (12) use constants to represent the net consumption/production terms. DO, ${\mathrm{DO}}_{\text{high}}$, and ${\mathrm{DO}}_{\mathrm{low}}$ are the DO level, the threshold for high DO levels (set at 50% in this work), and the threshold for low DO levels (set at 10% in this work), respectively. It should be noted that these thresholds are set in line with the available experimental datasets. Further experiments with more DO levels would be needed to create higher‐resolution models for the impact of DO.

$Q_{p}$ was described as proportional to the changes in $\left(\mu -\mu_{d}\right)$ in Equation (13), where $q_{p}$ is a constant and $\left(\mu -\mu_{d}\right)$ is positive. In cases where $\left(\mu -\mu_{d}\right)$ is negative, the term is set to zero.

Additionally, the entire model was updated to account for the impacts of lactate consumption, for example, by considering a positive effect of lactate concentration levels on μ as previously reported.^36^ On the other hand, at the net lactate consumption stages ($Q_{\mathrm{lac}}<0$), an enhancement to cell growth was assumed as a result of its use as a nutrient. Therefore, two decision points were set for the determination of model parameters: (1) depending on the value of DO, $Q_{\mathrm{lac}}$ and $Q_{\mathrm{gln}}$ are calculated as either a function of glutamine concentrations (at high DO levels) or as constants (Equations (11) and (12)); (2) μ is calculated as different functions of lactate and glutamine concentrations as shown in Equation (14). In this work, ${\mathrm{DO}}_{\text{high}}$ was set as 50% and ${\mathrm{DO}}_{\mathrm{low}}$ was set as 10% based on the applied experimental strategy and the analysis in the previous sections. Different parameters ${\mathrm{KI}}_{\mathrm{lac}1}$ and ${\mathrm{KI}}_{\mathrm{lac}2}$ were introduced at different DO levels to consider the potential changes in the lactate roles on μ before and after the DO shift. For $\mu_{d}$, no differentiation was assumed as shown in Equation (15).

(14) $$\mu =\{\begin{array}{l} {\mu_{\max1}\left(\frac{\left[\mathrm{GLC}\right]}{K_{\mathrm{glc}}+\left[\mathrm{GLC}\right]}\right)\left\{\frac{1}{1+\exp\left(-\frac{\left[\mathrm{GLN}\right]}{K_{\mathrm{gln}}}\right)}\right\}\left(\frac{{\mathrm{KI}}_{\mathrm{lac}1}}{{\mathrm{KI}}_{\mathrm{lac}1}+\left[\mathrm{LAC}\right]}\right)|}_{\mathrm{DO}={\mathrm{DO}}_{\text{high}}\cap Q_{\mathrm{lac}}>0}\\ {\mu_{\max1}\left(\frac{\left[\mathrm{GLC}\right]}{K_{\mathrm{glc}}+\left[\mathrm{GLC}\right]}\right)\left(\frac{\left[\mathrm{LAC}\right]}{K_{\mathrm{lac}}+\left[\mathrm{LAC}\right]}\right)|}_{\mathrm{DO}={\mathrm{DO}}_{\text{high}}\cap Q_{\mathrm{lac}}\leq 0}\\ {\mu_{\max1}\left(\frac{\left[\mathrm{GLC}\right]}{K_{\mathrm{glc}}+\left[\mathrm{GLC}\right]}\right)\left(\frac{{\mathrm{KI}}_{\mathrm{lac}2}}{{\mathrm{KI}}_{\mathrm{lac}2}+\left[\mathrm{LAC}\right]}\right)|}_{\mathrm{DO}={\mathrm{DO}}_{\mathrm{low}}} \end{array}$$

(15) $$\mu_{d}=k_{d}\left(\frac{\left[\mathrm{LAC}\right]}{{\mathrm{KD}}_{\mathrm{lac}}+\left[\mathrm{LAC}\right]}\right)\left(\frac{\left[\mathrm{AMM}\right]}{{\mathrm{KD}}_{\mathrm{amm}}+\left[\mathrm{AMM}\right]}\right)\left(\frac{{\mathrm{KDI}}_{\mathrm{glc}}}{{\mathrm{KDI}}_{\mathrm{glc}}+\left[\mathrm{GLC}\right]}\right)$$

In Equations (14) and (15), $K_{\mathrm{gln}}$ is the threshold glutamine concentration to fulfill one cell's glutamine requirement. K, KI, KD, and KDI are the Monod model parameters.

Experiments (A) and (B) were conducted in shaken tanks, while Experiment (C) was conducted in a stirred tank. This could lead to different mixing properties affecting the mass transfer and cell metabolism inside the reactors in the three experiments. Stirred tanks have mixing blades, leading to a faster mass transfer and the achievement of homogeneous environments, however, stronger shear stresses are expected. In shaken tanks, mass transfer limitations can exacerbate the difference between bulk and cell‐surface concentrations. Such differences may necessitate updating the model to account for the mass transfer limitations in shaken tanks.

At low DO levels, all experiments showed lower observed $\left(\mu -\mu_{d}\right)$ compared to higher DO levels (Figure 4). However, different trends in lactate concentrations were observed in the different reactor types especially at low DO levels (Figure 3 **(i)**). Therefore, Equations (14) and (15) were updated to better fit the shaken tank experiments as shown in Equations (16) and (17). The modifications to the equations reflect the difference in the impact of lactate concentrations on cell growth and death in the low DO region. Different parameter values were assumed at each DO level, whereby $\mu_{\max2}$, $k_{d2}$ and ${\mathrm{KD}}_{\mathrm{lac}2}$ represent the parameter values at low DO levels in Equations (16) and (17).

(16) $$\mu =\{\begin{array}{l} {\mu_{\max1}\left(\frac{\left[\mathrm{GLC}\right]}{K_{\mathrm{glc}}+\left[\mathrm{GLC}\right]}\right)\left\{\frac{1}{1+\exp\left(-\frac{\left[\mathrm{GLN}\right]}{K_{\mathrm{gln}}}\right)}\right\}\left(\frac{{\mathrm{KI}}_{\mathrm{lac}1}}{{\mathrm{KI}}_{\mathrm{lac}1}+\left[\mathrm{LAC}\right]}\right)|}_{\mathrm{DO}={\mathrm{DO}}_{\text{high}}\cap Q_{\mathrm{lac}}>0}\\ {\mu_{\max1}\left(\frac{\left[\mathrm{GLC}\right]}{K_{\mathrm{glc}}+\left[\mathrm{GLC}\right]}\right)\left(\frac{\left[\mathrm{LAC}\right]}{K_{\mathrm{lac}}+\left[\mathrm{LAC}\right]}\right)|}_{\mathrm{DO}={\mathrm{DO}}_{\text{high}}\cap Q_{\mathrm{lac}}\leq 0}\\ {\mu_{\max2}\left(\frac{\left[\mathrm{GLC}\right]}{K_{\mathrm{glc}}+\left[\mathrm{GLC}\right]}\right)\left(\frac{{\mathrm{KI}}_{\mathrm{lac}2}}{{\mathrm{KI}}_{\mathrm{lac}2}+\left[\mathrm{LAC}\right]}\right)|}_{\mathrm{DO}={\mathrm{DO}}_{\mathrm{low}}} \end{array}$$

(17) $$\mu_{d}=\left\{\begin{array}{c} {\left.k_{d1}\left(\frac{\left[\mathrm{LAC}\right]}{{\mathrm{KD}}_{\mathrm{lac}1}+\left[\mathrm{LAC}\right]}\right)\left(\frac{\left[\mathrm{AMM}\right]}{{\mathrm{KD}}_{\mathrm{amm}}+\left[\mathrm{AMM}\right]}\right)\left(\frac{{\mathrm{KDI}}_{\mathrm{glc}}}{{\mathrm{KDI}}_{\mathrm{glc}}+\left[\mathrm{GLC}\right]}\right)\;\right|}_{\mathrm{DO}={\mathrm{DO}}_{\text{high}}}\\ {\left.k_{d2}\left(\frac{\left[\mathrm{LAC}\right]}{{\mathrm{KD}}_{\mathrm{lac}2}+\left[\mathrm{LAC}\right]}\right)\left(\frac{\left[\mathrm{AMM}\right]}{{\mathrm{KD}}_{\mathrm{amm}}+\left[\mathrm{AMM}\right]}\right)\left(\frac{{\mathrm{KDI}}_{\mathrm{glc}}}{{\mathrm{KDI}}_{\mathrm{glc}}+\left[\mathrm{GLC}\right]}\right)\;\right|}_{\mathrm{DO}={\mathrm{DO}}_{\mathrm{low}}} \end{array}\right.$$

The shaken‐tank model was fit to Experiment (A), and then validated with Experiment (B). The stirred‐tank model was fit to Experiment (C). More datasets from the stirred‐tank reactor are still needed to fully validate the model. However, the obtained results and model fit can still be useful for developing a deeper understanding of the differences between the performance in both reactor types. The Nelder–Mead method was used to maximize the sum of $R^{2}$ between calculated and measured values of all system components. To analyze the uncertainty of the estimated parameters, standard errors were calculated by inverting the Hessian matrix. The python package, lmfit was used for the calculation.

## 3 RESULTS AND DISCUSSION

### 3.1 Modeling results

**FIGURE 5:**
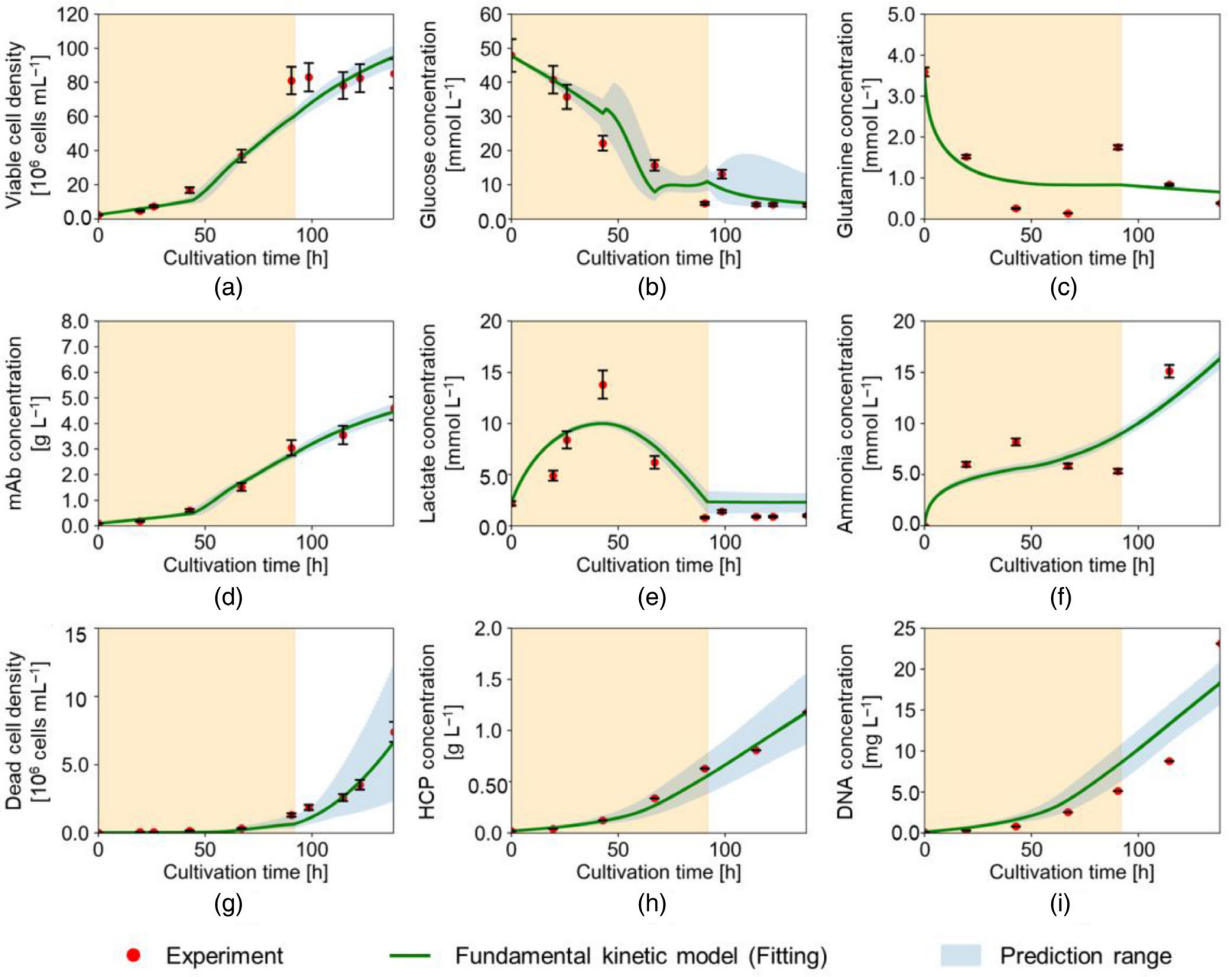
Fitting results to Experiment (A) with the proposed model: density/concentration of (a) viable cell, (b) glucose, (c) glutamine, (d) mAb, (e) lactate, (f) ammonia, (g) dead cell, (h) HCP, (i) DNA. Red dots represent the experimental data. Solid green lines represent the simulated results. Shaded areas represent high dissolved oxygen (${\mathrm{DO}}_{\text{high}}$) levels.

**FIGURE 6:**
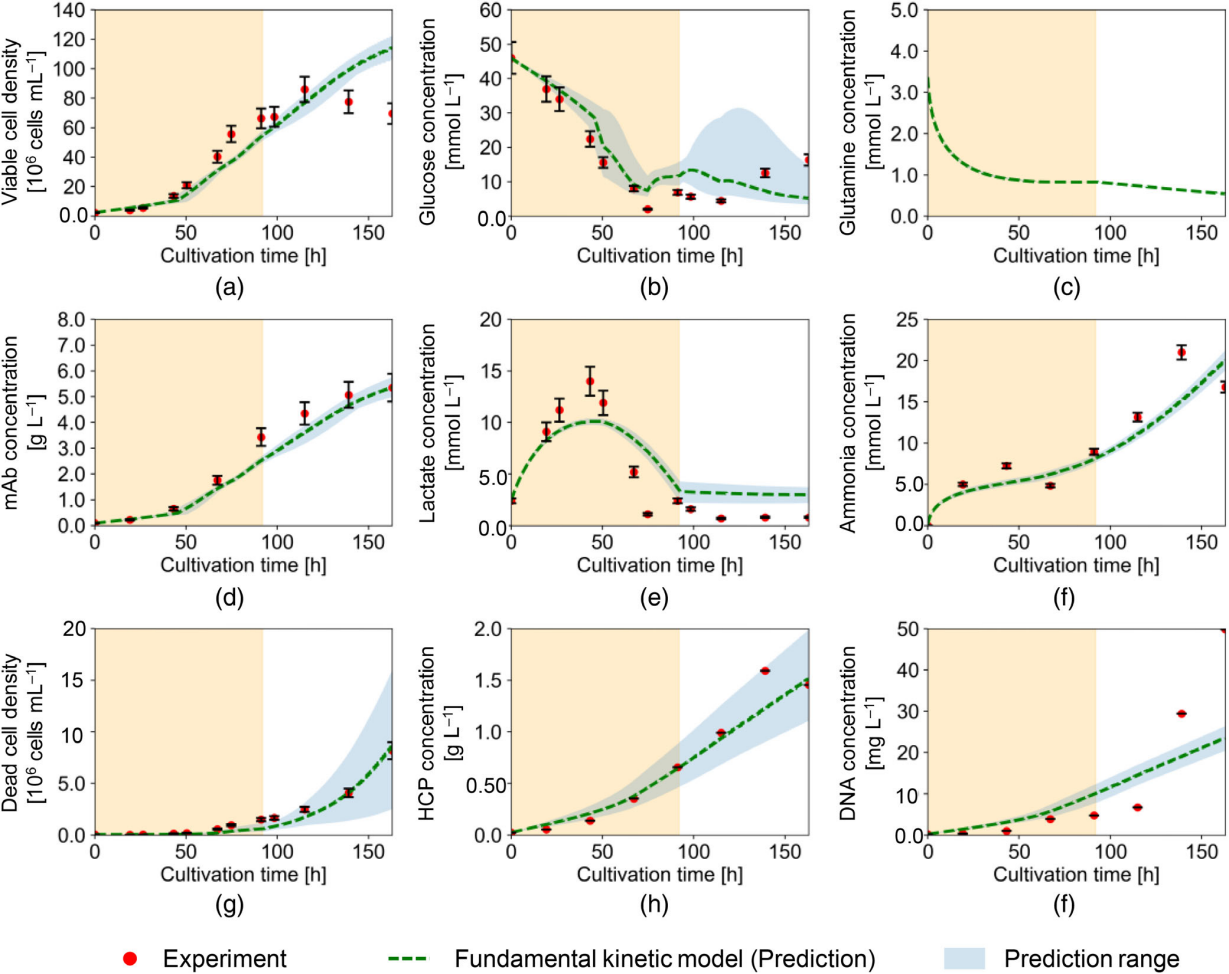
Validation results to Experiment (B) with the proposed model: density/concentration of (a) viable cell, (b) glucose, (c) glutamine, (d) mAb, (e) lactate, (f) ammonia, (g) dead cell, (h) HCP, (i) DNA. Red dots represent the experimental data. Dotted green lines represent the simulated results with the same parameters as Experiment (A). Shaded areas represent high dissolved oxygen (${\mathrm{DO}}_{\text{high}}$) levels.

**FIGURE 7:**
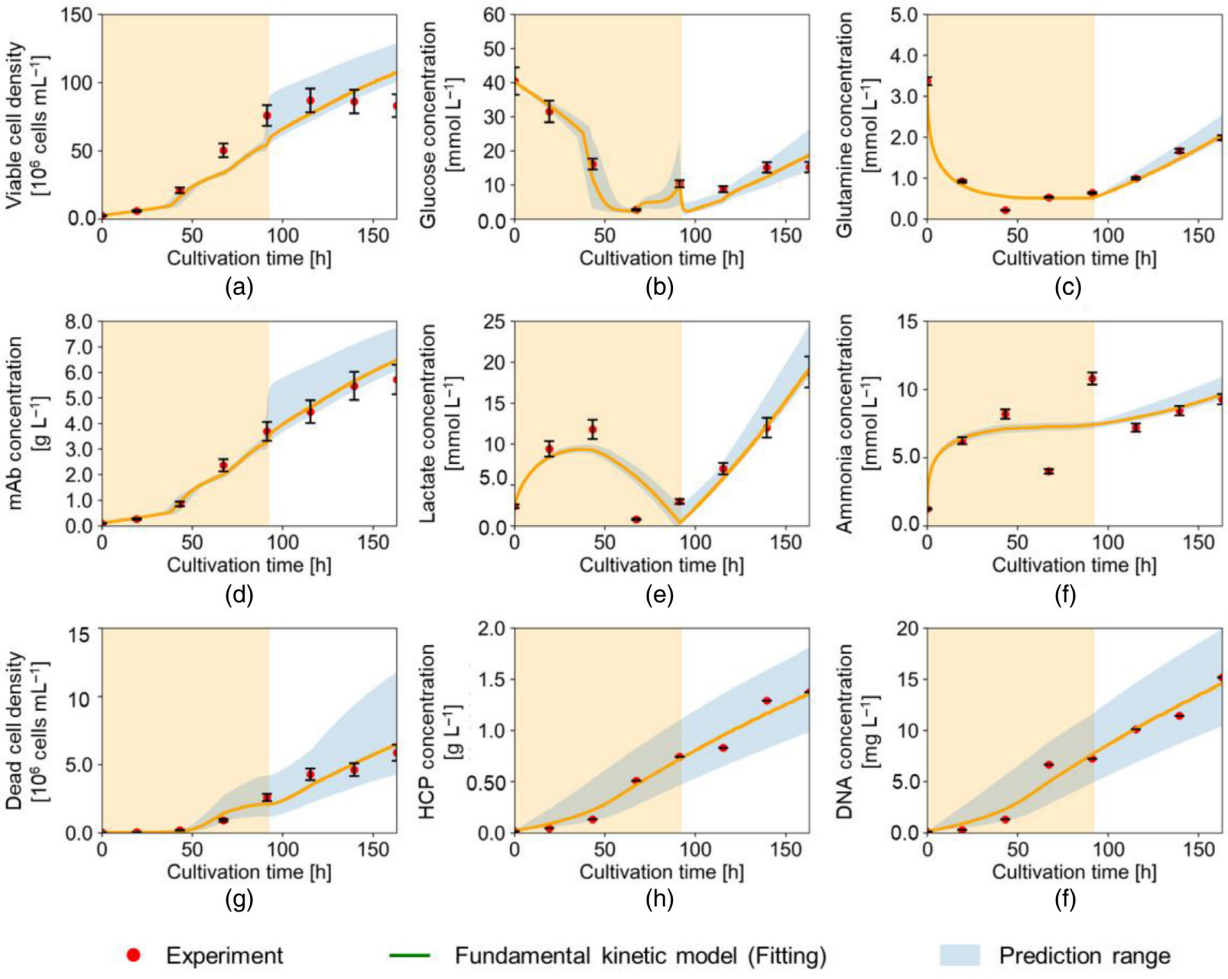
Fitting results to Experiment (c) with the proposed model: density/concentration of (a) viable cell, (b) glucose, (c) glutamine, (d) mAb, (e) lactate, (f) ammonia, (g) dead cell, (h) HCP, (i) DNA. Red dots represent the experimental data. Solid orange lines represent the simulated results. Shaded areas represent high dissolved oxygen (${\mathrm{DO}}_{\text{high}}$) levels.

Figures 5 and 6 show the experimental results obtained in shaken‐tank reactors, Experiments (A) and (B), respectively. The error bars reflect the measurement error for each component. The figures also show the results of the model fitting and validation. The prediction range represents the range between the minimum and maximum predicted profiles using the estimated parameter values and the standard errors assuming a normal distribution of the model parameter values. In Figure 5, the solid green line shows the results of fitting the model to the results from Experiment (A); The dotted green line in Figure 6 shows the results of predicting the performance in Experiment (B) using the parameters estimated from Experiment (A). Figure 7 shows the experimental results obtained for Experiment (C) with the stirred‐tank reactor cultivation and the results of the model fitting. It is worth noting that, as planned, the change in DO seems to be directly connected with the suppression of the growth phase and the attainment of the stationary phase in all three experiments. The experimental results shown in Figures 5 and 6 confirm that the biggest difference in the shaken‐tank experiments compared to that of the stirred tank in Figure 7 lies in the lactate profiles at the low DO level (t>92h). Additionally, in Experiments (A) and (B), ammonia and DNA concentrations increased more sharply at the low DO level than in Experiment (C). This result suggests that the differences between the shaken‐ and stirred‐tank reactors are more pronounced at the lower DO level.

Table 2 presents the coefficients of determination $R^{2}$ for the calculated concentrations with each experiment. Table 3 shows the estimated parameters for Experiments (A) and (C) with their standard error and the percentage change in parameter values between the two experiments with different reactor types. The results shown in Figure 5 and Table 2 confirm that the model fits well with cultivation in the shaken‐tank reactor with an average $R^{2}$ of 0.884. The change in the estimated parameter values from ($k_{d1}$, ${\mathrm{KD}}_{\mathrm{lac}1}$) to ($k_{d2},{\mathrm{KD}}_{\mathrm{lac}2}$) showed that the model differentiated the impact of bulk lactate concentrations on $\mu_{d}$ at different DO levels (Table 3).

**TABLE 2:** Coefficient of determination $R^{2}$ for calculated concentration profiles. Experiments (a) and (C) values show the fitting accuracy. The prediction accuracy of Experiment (b) is given using the parameters estimated in Experiment (a).

	Orbitally‐shaken tank		Stirred tank
	Experiment (A)	Experiment (B)	Experiment (C)
	Fitting	Prediction	Fitting
Viable cell	0.929	0.707	0.841
Glucose	0.918	0.832	0.953
Glutamine	0.788	^†^	0.976
mAb	0.990	0.950	0.973
Lactate	0.772	0.674	0.835
Ammonia	0.747	0.831	0.633
Dead cell	0.966	0.955	0.955
HCP	0.987	0.947	0.979
DNA	0.861	0.606	0.974

^†^ Experimental data and calculated results could not be compared because glutamine concentration was not measured in Experiment (B).

**TABLE 3:** Estimated parameters of Experiments (a) and (c) with standard error. Percentage changes in parameter values from Experiment (c) relative to the values for Experiment (a) are shown. Shaded cells represent parameters with a difference larger than ±35% between the two experiments divided into different categories indicated by color: in pink, Monod constants related to growth rate; in orange, Monod constants related to death rate; in yellow, glutamine consumption and lactate production at low DO level; in green, ammonia production rate constant independent of glutamine consumption; in blue, constants related to dead cell lysis.

Parameter	Unit	Experiment (A)	Experiment (C)	Percentage change
$\mu_{\max1}$	h^−1^	0.271 ± 1.16 × 10^−2^	0.282 ± 1.29 × 10^−2^	4.06%
$\mu_{\max2}$	h^−1^	0.296 ± 6.72 × 10^−2^	−	−
$k_{d1}$	h^−1^	5.15 × 10^−2^ ± 1.33 × 10^−2^	4.49 × 10^−2^ ± 7.60 × 10^−3^	−12.8%
$k_{d2}$	h^−1^	3.15 × 10^−2^ ± 9.40 × 10^−3^	−	−
$K_{\mathrm{glc}}$	mmol L^−1^	36.7 ± 2.38	14.1 ± 1.50	−61.6%
$K_{\mathrm{gln}}$	mmol L^−1^	0.631 ± 0.182	0.693 ± 0.200	9.83%
$K_{\mathrm{lac}}$	mmol L^−1^	6.46 ± 1.61	6.36 ± 2.61	−1.55%
${\mathrm{KI}}_{\mathrm{lac}1}$	mmol L^−1^	3.87 ± 0.503	2.70 ± 0.234	−30.2%
${\mathrm{KI}}_{\mathrm{lac}2}$	mmol L^−1^	1.15 ± 0.469	1.16 ± 0.153	0.870%
${\mathrm{KD}}_{\mathrm{lac}1}$	mmol L^−1^	12.6 ± 5.29	9.61 ± 2.69	−23.7%
${\mathrm{KD}}_{\mathrm{lac}2}$	mmol L^−1^	^†^	−	−
${\mathrm{KD}}_{\mathrm{amm}}$	mmol L^−1^	6.96 ± 2.22	6.03 ± 1.62	−13.4%
${\mathrm{KDI}}_{\mathrm{glc}}$	mmol L^−1^	0.582 ± 0.103	0.806 ± 0.158	38.5%
$Y_{X_{v}/\mathrm{glc}}$	cells mmol^−1^	4.89 × 10^8^ ± 2.37 × 10^7^	4.83 × 10^8^ ± 2.22 × 10^7^	−1.23%
$m_{\mathrm{glc}}$	mmol cell^−1^ h^−1^	^†^	^†^	^†^
$k_{\mathrm{gln}11}$	mmol cell^−1^ h^−1^	1.21 × 10^−12^ ± 4.13 × 10^−19^	1.60 × 10^−12^ ± 1.51 × 10^−18^	32.2%
$k_{\mathrm{gln}12}$	−	^†^	^†^	^†^
$k_{\mathrm{gln}13}$	L mmol^−1^	1.67 ± 6.70 × 10^−3^	2.03 ± 1.50 × 10^−2^	21.6%
$k_{\mathrm{gln}14}$	mmol cell^−1^ h^−1^	−4.88 × 10^−12^ ± 6.93 × 10^−22^	−4.56 × 10^−12^ ± 3.89 × 10^−22^	−6.56%
$k_{\mathrm{gln}21}$	mmol cell^−1^ h^−1^	1.57 × 10^−14^ ± 6.19 × 10^−17^	−3.07 × 10^−13^ ± 2.91 × 10^−17^	^‡^
$k_{\mathrm{lac}11}$	mmol cell^−1^ h^−1^	2.63 × 10^−11^ ± 4.08 × 10^−15^	2.83 × 10^−11^ ± 1.17 × 10^−14^	7.61%
$k_{\mathrm{lac}12}$	−	6.50 × 10^−2^ ± 1.43 × 10^−3^	4.61 × 10^−2^ ± 2.55 × 10^−3^	−29.1%
$k_{\mathrm{lac}13}$	L mmol^−1^	1.19 ± 5.25 × 10^−3^	1.38 ± 1.16 × 10^−2^	−10.6%
$k_{\mathrm{lac}14}$	mmol cell^−1^ h^−1^	−6.41 × 10^−11^ ± 2.28 × 10^−14^	−5.73 × 10^−11^ ± 5.10 × 10^−14^	16.0%
$k_{\mathrm{lac}21}$	mmol cell^−1^ h^−1^	8.85 × 10^−14^ ± 6.50 × 10^−16^	3.50 × 10^−12^ ± 2.44 × 10^−15^	^‡^
$k_{\mathrm{amm}1}$	−	1.83 ± 0.125	2.01 ± 8.66 × 10^−2^	9.84%
$k_{\mathrm{amm}2}$	mmol cell^−1^ h^−1^	2.49 × 10^−12^ ± 1.00 × 10^−15^	7.35 × 10^−13^ ± 1.03 × 10^−16^	−70.5%
$q_{p}$	g cell^−1^	4.71 × 10^−11^ ± 1.82 × 10^−13^	6.07 × 10^−11^ ± 2.75 × 10^−13^	28.9%
$K_{\text{ldcell}}$	cells L^−1^	1.29 × 10^8^ ± 2.91 × 10^7^	7.46 × 10^7^ ± 6.26 × 10^7^	−42.2%
$K_{\mathrm{dh}}$	mg L^−1^	^†^	^†^	^†^
$K_{\mathrm{dd}}$	mg L^−1^	^†^	^†^	^†^
$r_{\text{dcell}}$	cells L^−1^ h^−1^	1.91 × 10^6^ ± 9.88 × 10^4^	1.36 × 10^6^ ± 1.92 × 10^5^	−28.8%
$r_{h}$	mg L^−1^ h^−1^	^†^	^†^	^†^
$r_{d}$	mg L^−1^ h^−1^	^†^	^†^	^†^
$Y_{\mathrm{hcp}/X_{d}}$	mg cell^−1^	9.09 × 10^−6^ ± 1.93 × 10^−6^	9.67 × 10^−6^ ± 1.76 × 10^−6^	6.38%
$Y_{\mathrm{dna}/X_{d}}$	mg cell^−1^	1.42 × 10^−7^ ± 7.46 × 10^−9^	1.04 × 10^−7^ ± 1.93 × 10^−8^	−26.8%

^†^ Indicates parameter values of negligible impact on the corresponding terms in the model, and the difference was not calculated.

^‡^ Indicates difference in parameter values between Experiments (a) and (C) were larger than 100%.

^−^ Dash: Indicates that the parameter was not used in the model corresponding to the reactor type.

The application of the parameters fitted to Experiment (A) generally yielded a high accuracy when used to predict the performance in Experiment (B), as shown in Figure 6. The accuracy of the fit to the glutamine concentrations could not be assessed due to the absence of glutamine concentration measurements in Experiment (B). An average goodness of fit of $R^{2}$ = 0.813 was achieved (see Table 2). HCP and mAb concentrations were among those best predicted with $R^{2}$ of 0.947 and 0.950, respectively. This can be reassuring for model use to predict process performance and product quality. The dead cell density was also well predicted with an $R^{2}$ value of 0.955. On the other hand, viable cell density and DNA concentration showed a larger deviation after 115 h until the end, which was not fully covered by the culture duration of Experiment (A). Regarding Experiment (C), Figure 7 and Table 2 confirm that the model fits well with cultivation in the stirred‐tank reactor with an average $R^{2}$ of 0.902.

Analysis of the estimated parameter values in Table 3 for both Experiments (A) and (C), shows that most parameters were calculated with a relatively narrow range of standard error (<10% on average in that group for both experiments). For each experiment, only a few terms had a wider range of standard error (i.e., >30%), ${\mathrm{KI}}_{\mathrm{lac}2}$, ${\mathrm{KD}}_{\mathrm{lac}}$, and ${\mathrm{KD}}_{\mathrm{amm}}$ in Experiment (A); and $K_{\mathrm{lac}}$ and $K_{\text{ldcell}}$ in Experiment (C). At higher DO levels, the first term in Equations (11) and (12) would be positive representing the specific lactate production and glutamine consumption rates, respectively. while the second term would be constant and negative, representing the specific lactate consumption and glutamine production rates, respectively. At lower DO levels, a difference was observed between Experiments (A) and (C) in terms of production/consumption trends. The values of $k_{\mathrm{lac}21}$ indicate a sharp increase in specific lactate production in Experiment (C) (2 orders of magnitude compared to Experiment (A)), whereas the values of $k_{\mathrm{gln}21}$ show that in Experiment (A), a very weak net consumption rate of glutamine was observed as opposed to net glutamine production in Experiment (C). The estimated value of $k_{\mathrm{amm}1}$ was approximately 2 in both experiments, which suggests that most of the glutamine is consumed in the TCA cycle.

The parameters where the values varied largely between Experiments (A) and (C) (see the shaded values in Table 3) can be classified into the following five categories. The first category is the Monod constants related to the specific growth rate ($K_{\mathrm{glc}}$, shaded in pink), where the change in values indicated a higher concentration of glucose was required to achieve the same specific growth rate as in Experiment (C). The second category is the Monod constants related to the specific death rate (${\mathrm{KDI}}_{\mathrm{glc}}$ shaded in orange). The change in the parameter values in this category indicated that the specific death rate was less sensitive to lower glucose concentrations in Experiment (A). The third category is the specific rates of glutamine consumption and lactate production at low DO levels ($k_{\mathrm{gln}21}$, and $k_{\mathrm{lac}21}$; shaded in yellow). The change indicated lower glutamine consumption and higher lactate production rates at the low DO levels in the stirred‐tank experiment. The fourth category is $k_{\mathrm{amm}2}$ (shaded in green), indicating a change in the specific ammonia production rate from sources other than glutamine. The fifth category includes the parameters ($K_{\text{ldcell}}$; shaded in blue) indicating a higher specific dead cell lysis rate.

The gentler mixing experienced in the shaken‐tank reactors could lead to less efficient mixing of system components, resulting in a bigger gap between bulk solution concentrations and those immediately surrounding the cells. This gap could explain the lower sensitivity to bulk nutrient concentrations, like in the case of glucose, and the higher sensitivities to lactate concentrations, which would be generated from the cells. The same mixing constraints could also explain the lower specific dead cell lysis rates ($K_{\text{ldcell}}$) in the shaken‐tank reactors. It should be noted that $K_{\text{ldcell}}$ also had a relatively large standard error (Table 3), which indicates that the representation of dead cell lysis can still be further studied. Generally, a higher cell death rate $\mu_{d}$ was calculated for Experiment (A), especially at the lower DO level. The release of ammonia from the dead cells could then have resulted in the higher observed values of ammonia generation rate ($k_{\mathrm{amm}2}$) from sources other than glutamine. At the lower DO level, higher glutamine and lactate concentrations were observed in Experiment (C) compared to (A), which could explain the partial metabolic shift back from lactate consumption to production.

Toward the end of Experiment (B), a higher concentration of ammonia was observed. The impact of such higher concentrations on the death rate in a shaken‐tank could have been underestimated by using the parameters from Experiment (A). A higher ${\mathrm{KD}}_{\mathrm{amm}}$ was observed in Experiment (A) compared to (C), which implied a lower sensitivity of the death rate to high ammonia concentrations. The true value of ${\mathrm{KD}}_{\mathrm{amm}}$ might lie between that of Experiments (A) and (C) and should be more carefully determined in future experiments. The deviation in the DNA concentrations was observed despite the accurate prediction of dead cell density and HCP concentrations. This indicates a phenomenon related to the release of DNA specifically. During model generation, the dead cell lysis and release of impurities (i.e., HCP and DNA) were assumed to occur concurrently. However, delays in the release of DNA can be explained by the differences in the source of the release. One possibility could be that DNA is mostly released from the cell nucleus while HCP is mostly released from inside the cell. This delay, which was not accounted for in the model, would be exacerbated by the weaker mixing in the shaken‐tank reactors. This could have led to the sharper increase in DNA concentrations observed toward the end of the experiment in Experiments (A) and (B). Experiment (A) was terminated after a shorter duration compared to Experiment (B), and therefore, the full extent of DNA release may not have been completely observed.

### 3.2 Value and limitations of the developed model

The developed model could describe the profiles of essential factors in cell cultivation, including both product and process‐related impurities in a simple manner, even in systems where shifts in lactate production and consumption occur. The approach elucidated that the impact of glutamine depletion on lactate metabolism changed based on the DO levels. The application of the model led to a higher understanding of the changes in cell metabolism with simultaneous changes in system component levels and elements of the cultivation environment.

The developed mechanistic model could be easily implemented to describe dynamic changes based on concentrations outside the cells. Therefore, it can be used for simulation and process evaluation to explore a range of wider operating conditions, for example, to assess different feeding strategies, than previous data‐driven or steady state models.^12^ Also, given the novelty of the CHO‐MK cell lines, more cell and process developments are still ongoing to further enhance its productivity. Having more representative models showing the high‐resolution effects of glutamine and ammonia, for example, is more in line with the needs of the actual process design.

Although the developed model achieved a good fit for both reactor types, more experiments are needed for validation especially for the stirred‐tank reactor. In addition, despite the good fit, some gaps between the experimental data and calculated results still exist. For example, the deviation observed in the DNA concentration profile in Experiment (B) indicates the necessity of updating the modeling assumption that both HCP and DNA will be released immediately after cell death and lysis. Also, it is still necessary to use different parameter sets to describe systems with different agitation methods (stirred tank vs. shaken tank). Analyzing the heterogeneity in the system and gaps of concentrations between bulk and cell surface would provide valuable insight toward incorporating this difference in agitation effects into the model. Despite these shortcomings, the developed model was able to elucidate the differences between kinetics in both reactors, which is worth further investigation to fully understand the optimal mixing settings with providing appropriate control measures to maximize the benefit from each reactor type. It is worth noting that an upward drift in pH was observed toward the end of Experiments (A) and (B), while in Experiment (C), the pH was more stable. No active pH control was applied during the experiments. In Experiments (A) and (B), the lactate concentrations stabilized at values close to zero in the second half of the experiments, while in Experiment (C), there was a rapid rise in lactate concentrations after the DO shift. Ammonia concentrations were also higher in Experiments (A) and (B) relative to Experiment (C) which contributed to the difference in pH profiles toward the end of the runs. While the differences between the mixing regimes reactors could account for the change in behavior between the shaken‐ and stirred‐tank reactors, the differences in the pH profiles could have further exacerbated the observed variations.

Furthermore, model uncertainty could still be reduced. Applying more in‐line PAT monitoring methods, particularly acting as soft sensors to concentration measurements, more accurate depictions of the timing of metabolic shifts could be achieved. Uncertainties related to measurement errors would be minimized and model applicability would be enhanced. While most model parameters could be determined with relatively narrow standard error, some parameters remain uncertain especially related to cell death, for example, $K_{\text{ldcell}}$. More data points within each experiment would generally contribute to achieving lower parameter uncertainty as well, for example regarding the quantification of dead cell lysis. In‐line PAT data could also be used in combination with the model results for more advanced model predictive control applications.

For more versatile and advanced model development toward applications in process design, experiments with diverse levels and timings of DO shifts might be helpful to achieve the required model resolution in each reactor type. Also, quantifying the relationship between DO and oxygen sparging rate would contribute to a better understanding of the impact of oxygen on the system. Based on these two factors and the volumetric mass transfer coefficient ($k_{L}a$) for oxygen, the oxygen uptake rate by cells can be calculated. The dynamics of oxygen uptake could lead to a deeper understanding of the cell phase shifts from the perspective of cell metabolism and contribute to the design of advanced DO control strategies.

## 4 CONCLUSIONS

In this work, we presented an unstructured mechanistic model depicting the cell metabolic shifts by incorporating nutrient dynamics and the impact of changes in DO levels. The model could describe well the profile of major component concentrations in experiments using the novel CHO‐MK 9E‐1 cell line in both stirred‐ and shaken‐tank reactors. The developed model can be used for the prediction of the performance of cell cultivation with different conditions, such as feeding and DO control strategies. The model would contribute to narrowing down the options in process development before conducting actual experiments. Further analysis can explore variations of the timing of the DO shifts, develop continuous equations as a function of DO levels, and investigate the relationship with the depletion of different nutrients, such as glutamine. Also, formulating the difference in mixing regimes between different agitation systems and the resulting relationship between DO and oxygen sparging rates are to be explored. It would lead to a deeper understanding of the dynamics of cell metabolism and oxygen consumption for model‐based design of control strategies.

## NOTATION

**Table:** 

μ	h^−1^	Specific cell growth rate
$\mu_{d}$	h^−1^	Specific cell death rate
$\mu_{\max1}$	h^−1^	Maximum specific cell growth rate for shaken tank at high DO and for stirred‐tank
$\mu_{\max2}$	h^−1^	Maximum specific cell growth rate for shaken tank at low DO
$\left[\mathrm{AMM}\right]$	mmol L^−1^	Ammonia concentration
$c_{\text{in}\_\mathrm{glc}}$	mmol L^−1^	Glucose concentration of the inlet (feed or perfusion) media
$c_{\text{suppl}}$	mmol L^−1^	Glucose concentration of the supplementary glucose solution
$c_{\text{in}\_\mathrm{gln}}$	mmol L^−1^	Glutamine concentration of the inlet (feed or perfusion) media
$\left[\mathrm{DNA}\right]$	mg L^−1^	DNA concentration
DO	%	Relative dissolved oxygen concentration to the saturation concentration at 37°C
${\mathrm{DO}}_{\text{high}}$	%	Threshold for high dissolved oxygen concentration levels
${\mathrm{DO}}_{\mathrm{low}}$	%	Threshold for low dissolved oxygen concentration levels
$F_{\text{bleed}}$	L h^−1^	Bleed flowrate
$F_{\text{harvest}}$	L h^−1^	Harvest flowrate
$F_{\text{in}}$	L h^−1^	Inlet flowrate
$F_{\text{suppl}}$	L h^−1^	Supplementary glucose flowrate
$\left[\mathrm{GLC}\right]$	mmol L^−1^	Glucose concentration
$\left[\mathrm{GLN}\right]$	mmol L^−1^	Glutamine concentration
$\left[\mathrm{HCP}\right]$	mg L^−1^	HCP concentration
$k_{\mathrm{amm}1}$	–	Ammonia production rate proportional to glutamine consumption
$k_{\mathrm{amm}2}$	mmol cell^−1^ h^−1^	Ammonia production rate proportional to other process parameters
$k_{d1}$	h^−1^	Maximum specific cell death rate for shaken tank at high DO and for stirred‐tank
$k_{d2}$	h^−1^	Maximum specific cell death rate for shaken tank at low DO
$K_{\mathrm{dd}}$	mg L^−1^	Constant for DNA dissolution
$K_{\mathrm{dh}}$	mg L^−1^	Constant for HCP dissolution
$K_{\mathrm{glc}}$	mmol L^−1^	Monod model parameter for μ
$K_{\mathrm{gln}}$	mmol L^−1^	Threshold glutamine concentration to fulfill one cell glutamine requirement
$k_{\mathrm{gln}11}$	mmol cell^−1^ h^−1^	Constant for $Q_{\mathrm{gln}}$
$k_{\mathrm{gln}12}$	–	Constant for $Q_{\mathrm{gln}}$
$k_{\mathrm{gln}13}$	L mmol^−1^	Constant for $Q_{\mathrm{gln}}$
$k_{\mathrm{gln}14}$	mmol cell^−1^ h^−1^	Constant for $Q_{\mathrm{gln}}$
$k_{\mathrm{gln}21}$	mmol cell^−1^ h^−1^	Constant for $Q_{\mathrm{gln}}$
$K_{\mathrm{lac}}$	mmol L^−1^	Monod model parameter for μ
$k_{\mathrm{lac}11}$	mmol cell^−1^ h^−1^	Constant for $Q_{\mathrm{lac}}$
$k_{\mathrm{lac}12}$	–	Constant for $Q_{\mathrm{lac}}$
$k_{\mathrm{lac}13}$	L mmol^−1^	Constant for $Q_{\mathrm{lac}}$
$k_{\mathrm{lac}14}$	mmol cell^−1^ h^−1^	Constant for $Q_{\mathrm{lac}}$
$k_{\mathrm{lac}21}$	mmol cell^−1^ h^−1^	Constant for $Q_{\mathrm{lac}}$
$K_{\text{ldcell}}$	cells L^−1^	Constant for dead cell lysis
${\mathrm{KD}}_{\mathrm{lac}1}$	mmol L^−1^	Monod model parameter for $\mu_{d}$ for shaken tank and stirred tank
${\mathrm{KD}}_{\mathrm{lac}2}$	mmol L^−1^	Monod model parameter for $\mu_{d}$ for shaken tank
${\mathrm{KD}}_{\mathrm{amm}}$	mmol L^−1^	Monod model parameter for $\mu_{d}$
${\mathrm{KDI}}_{\mathrm{glc}}$	mmol L^−1^	Monod model parameter for $\mu_{d}$
${\mathrm{KI}}_{\mathrm{lac}1}$	mmol L^−1^	Monod model parameter for μ
${\mathrm{KI}}_{\mathrm{lac}2}$	mmol L^−1^	Monod model parameter for μ
$\left[\mathrm{LAC}\right]$	mmol L^−1^	Lactate concentration
$m_{\mathrm{glc}}$	mmol cell^−1^ h^−1^	Glucose consumption for cell maintenance
P	g L^−1^	mAb concentration
$Q_{\mathrm{gln}}$	mmol cell^−1^ h^−1^	Specific glutamine consumption rate
$Q_{\mathrm{lac}}$	mmol cell^−1^ h^−1^	Specific lactate production rate
$Q_{p}$	g cell^−1^ h^−1^	Specific mAb production rate
$q_{p}$	g cell^−1^	Constant for $Q_{p}$
$r_{d}$	mg L^−1^ h^−1^	DNA dissolution rate
$r_{\text{dcell}}$	cells L^−1^ h^−1^	Dead cell lysis rate
$r_{h}$	mg L^−1^ h^−1^	HCP dissolution rate
t	h	Cultivation time
V	L	Solution volume
$X_{d}$	cells L^−1^	Dead cell density
$X_{v}$	cells L^−1^	Viable cell density
$Y_{\mathrm{dna}/X_{d}}$	mg cell^−1^	Specific mass released from a dead cell of DNA (DNA yield on dead cell lysis)
$Y_{\mathrm{hcp}/X_{d}}$	mg cell^−1^	Specific mass released from a dead cell of HCP (HCP yield on dead cell lysis)
$Y_{X_{v}/\mathrm{glc}}$	cells mmol^−1^	Glucose consumption for cell growth (Growth yield on glucose)

## AUTHOR CONTRIBUTIONS

**Kozue Okamura:** Conceptualization; investigation; writing – original draft; methodology; validation; visualization; writing – review and editing; formal analysis; data curation; software. **Sara Badr:** Conceptualization; methodology; formal analysis; validation; writing – review and editing; supervision. **Yusuke Ichida:** Conceptualization; methodology; investigation. **Akira Yamada:** Methodology; data curation; writing – review and editing. **Hirokazu Sugiyama:** Conceptualization; methodology; writing – review and editing; resources; supervision; project administration; funding acquisition.

## FUNDING INFORMATION

The Japan Agency for Medical Research and Development (AMED) [grant nos. JP21ae0121015, JP21ae0121016].

## CONFLICT OF INTEREST STATEMENT

The authors declare no conflict of interest.

## Supporting information


**Table S1:** Measurement devices.

## Data Availability

The data that support the findings of this study are available from the corresponding author upon reasonable request.
