# Supplementary material for: Modeling of cell cultivation for monoclonal antibody production processes considering lactate metabolic shifts
Source: Biotechnol Prog. 2024 Jun 24;40(6):e3486. doi: 10.1002/btpr.3486 (PMC11659809; doi:10.1002/btpr.3486)
Supplement: Supplementary file 1 — Table S1: Measurement devices. [file BTPR-40-e3486-s001.docx]

**Supporting Information**

**Modeling of cell cultivation for monoclonal antibody production processes considering metabolic shifts**

Kozue Okamura^1^, Sara Badr^1*^, Yusuke Ichida^1^, Akira Yamada^1^, Hirokazu Sugiyama^1^

^1^*Department of Chemical System Engineering, The University of Tokyo, 7-3-1, Hongo, Bunkyo-ku, 113-8656, Tokyo, Japan*

**Correspondence:*

TEL & FAX: +81 3 5841 6876

Email: [badr@pse.t.u-tokyo.ac.jp](mailto:badr@pse.t.u-tokyo.ac.jp)

Table S1. Measurement devices.

| Experiment | Measurement target | Device | Company |
| --- | --- | --- | --- |
| Experiment (A) | Viable cells | Vi-CELL XR | Beckman Coulter |
|  | Viability | Vi-CELL XR | Beckman Coulter |
|  | Glucose | GEM Premier 4000 | Werfen |
|  | Glutamine | LCMS8060 | Shimadzu |
|  | Lactate | GEM Premier 4000 | Werfen |
|  | Ammonia | Pocket Chem BA PA-4140 | arkray |
|  | mAb | Octet Qke | ForteBio |
|  | HCP | SpectraMax340PC384 | Molecular Devices |
|  | DNA | 7500 Fast Real-Time PCR System | Thermo Fisher |
|  | Dissolved oxygen | DO sensor (INPRO6800/12/220) | METTLER TOLEDO |
|  | pH | pH sensor (InPro3253/225PT1000) | METTLER TOLEDO |
|  | Agitation rate | Attached to the bioreactor | FUJIMORI KOGYO |
|  | Temperature | Attached to the bioreactor | FUJIMORI KOGYO |
|  | Air flow rate into gas phase | Attached to the bioreactor | FUJIMORI KOGYO |
|  | Oxygen sparging rate | Attached to the bioreactor | FUJIMORI KOGYO |
|  | Solution volume | Attached to the bioreactor | FUJIMORI KOGYO |
| Experiment (B) | Viable cells | Vi-CELL XR | Beckman Coulter |
|  | Viability | Vi-CELL XR | Beckman Coulter |
|  | Glucose | GEM Premier 4000 | Werfen |
|  | Lactate | GEM Premier 4000 | Werfen |
|  | Ammonia | Pocket Chem BA PA-4140 | arkray |
|  | mAb | Octet Qke | ForteBio |
|  | HCP | SpectraMax340PC384 | Molecular Devices |
|  | DNA | 7500 Fast Real-Time PCR System | Thermo Fisher |
|  | Dissolved oxygen | DO sensor (INPRO6800/12/220) | METTLER TOLEDO |
|  | pH | pH sensor (InPro3253/225PT1000) | METTLER TOLEDO |
|  | Agitation rate | Attached to the bioreactor | FUJIMORI KOGYO |
|  | Temperature | Attached to the bioreactor | FUJIMORI KOGYO |
| Experiment (B) | Air flow rate into gas phase | Attached to the bioreactor | FUJIMORI KOGYO |
|  | Oxygen sparging rate | Attached to the bioreactor | FUJIMORI KOGYO |
|  | Solution volume | Attached to the bioreactor | FUJIMORI KOGYO |
| Experiment (C) | Viable cells | Vi-CELL XR | Beckman Coulter |
|  | Viability | Vi-CELL XR | Beckman Coulter |
|  | Glucose | GEM Premier 4000 | Werfen |
|  | Glutamine | LCMS8060 | Shimadzu |
|  | Lactate | GEM Premier 4000 | Werfen |
|  | Ammonia | Pocket Chem BA PA-4140 | arkray |
|  | mAb | Octet Qke | ForteBio |
|  | HCP | SpectraMax340PC384 | Molecular Devices |
|  | DNA | 7500 Fast Real-Time PCR System | Thermo Fisher |
|  | Dissolved oxygen | DO sensor (INPRO6800/12/220) | METTLER TOLEDO |
|  | pH | pH sensor (InPro3253/225PT1000) | METTLER TOLEDO |
|  | Agitation rate | Attached to the bioreactor | Cytiva |
|  | Temperature | Attached to the bioreactor | Cytiva |
|  | Air flow rate into gas phase | Attached to the bioreactor | Cytiva |
|  | Oxygen sparging rate | Attached to the bioreactor | Cytiva |
|  | Solution volume | Attached to the bioreactor | Cytiva |
